# Sound waves for solving the problem of recrystallization in cryopreservation

**DOI:** 10.1038/s41598-023-34681-z

**Published:** 2023-05-10

**Authors:** Enrique Alcalá, Laura Encabo, Fatima Barroso, Adriana Puentes, Isabel Risco, Ramon Risco

**Affiliations:** 1 Escuela Superior de Ingenieria, C/Camino de los Descubrimientos s/n, University of Seville, 41092 Seville, Spain; 2 SafePreservation, C/Avda. De la Ciencias 55, 41020 Seville, Spain; 3 National Accelerators Centre-US, JA, CSIC, C/Tomas Alva Edison 7, 41092 Seville, Spain

**Keywords:** Biological techniques, Medical research

## Abstract

Organ biobanking is the pending subject of cryopreservation. Although the problem is multifaceted, advances in recent decades have largely related it to achieving rapid and uniform rewarming of cryopreserved samples. This is a physical challenge largely investigated in past in addition to cryoprotectant toxicity studies, which have also shown a great amount of advancement. This paper presents a proof-of-principle, based on the nematode *Caenorhabditis elegans*, of a technology capable of performing such a function: high intensity focused ultrasound. Thus, avoiding the problem of recrystallization, this worm, in its adult state, preserved at − $80\;^\circ{\rm C}$, has been systematically brought back to life after being heated with High Intensity Focused Ultrasound (HIFU) waves. The great advantage of this technology is that it is scalable; in addition, rewarming can be monitored in real time by MRI thermography and can be controlled by acoustic interferometry. We anticipate that our findings are the starting point for a possible approach to rewarming that can be used for cryopreservation of millimeter scale systems: either alone or in combination with other promising ways of heating, like nanowarming or dielectric heating, the present technology provides new ways of solving the physical aspects of the problem of recrystallization in cryopreservation, opening the door for the long-term storage of larger samples.

## Introduction

The preservation of organs in banks at low temperatures offers innumerable opportunities^1,2^. Currently, this possibility has remained elusive, with only some partial and isolated successes, i.e. in the rabbit kidney, sheep ovary or liver^3–5^. Although there are different cryopreservation strategies, for long-term cryogenic storage of organs, the damage caused by the eventual appearance of ice crystals is largely responsible for this situation. In the following paragraphs we will try to frame the problem within the general context of cryopreservation. Next, we will understand why rapid and uniform rewarming manages to avoid this. Finally, we will see how high intensity focused ultrasound is able to offer the solution.

**Figure 1:**
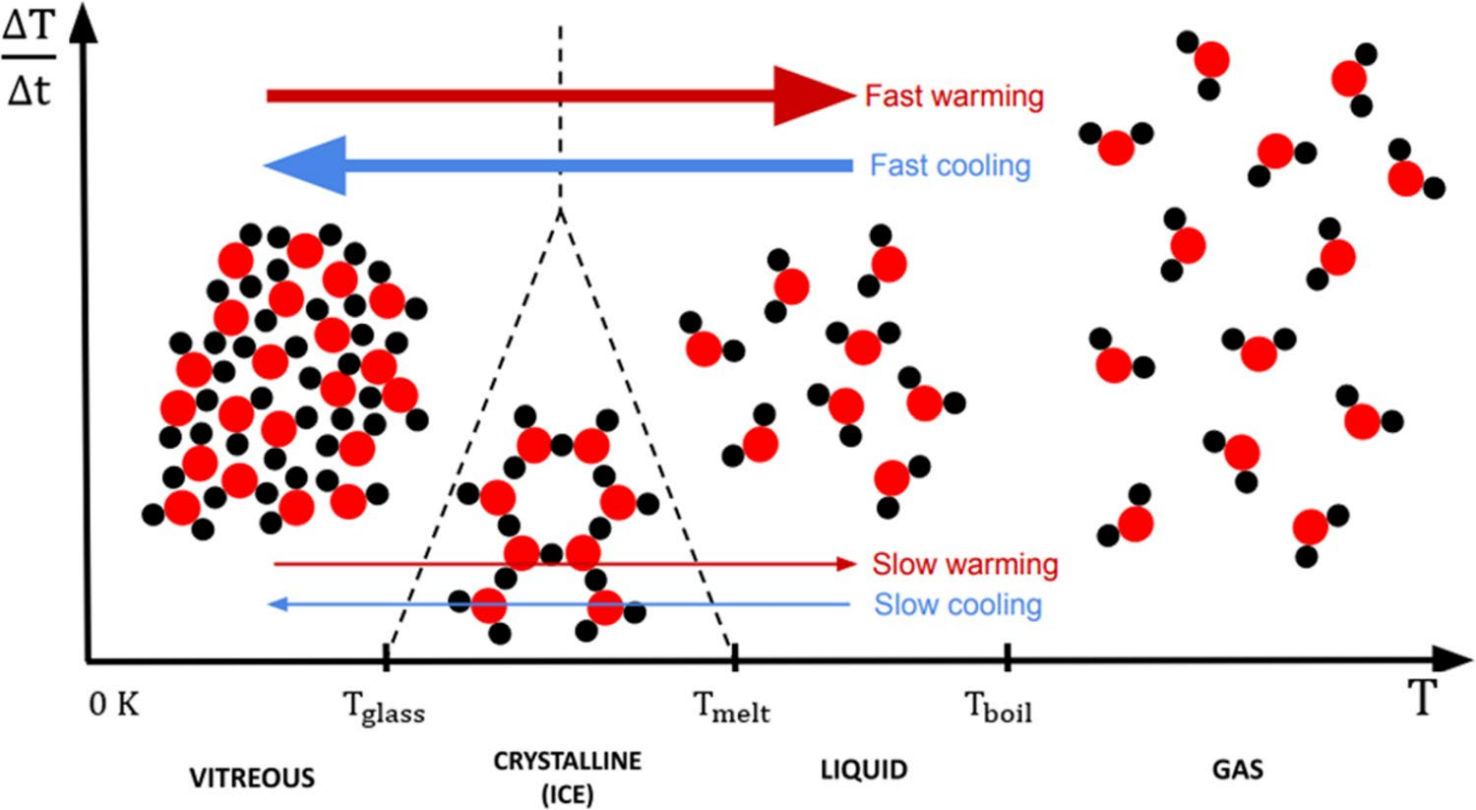
Effects of cooling and warming rates on the phase change for an aqueous system. The horizontal axis represents the temperature in Kelvin, with the transition temperatures between the four states (glass, crystal, liquid, and gas) labeled as and T_glass_ (glass transition temperature), T_melt_ (melting temperature) and T_boil_ (vaporization temperature). This axis can be traversed in both directions, corresponding to the changes of state when crossing the marked temperatures. The vertical axis represents the speed at which the temperature change occurs. The arrangement of the molecules of the system for the four mentioned states are represented in an illustrative manner. In this diagram, the speed at which it crosses through the marked temperatures is of special interest, the most relevant for the topic that concerns us being the change from liquid to glassy state. An excessively slow temperature change (low values on the vertical axis) to go from the liquid to the glassy phase, and vice versa, implies the necessary passage through the crystalline phase, which in aqueous systems implies the formation of ice. Instead, high cooling and/or warming rates (high values on the vertical axis) bypass the "crystalline" region, giving a direct change between the liquid and glassy states.

Already in 1940 Luyet^6^ understood well that the glassification of biological systems was possible simply by crossing the zone where ice could appear at a sufficient speed. This is represented symbolically in Fig. 1: when the temperature rises or falls, we go from one phase to another; however, we can skip a certain phase if the temperature change is fast enough. Thus, we can go from liquid to glass, and vice versa, without going through the crystalline state; for this, it is enough that the characteristic time of this transition is less than the characteristic time required for the nucleation and growth of the ice.

The problem Luyet ran into was that such cooling and warming rates were often impossible to reach in systems larger than a few tens of microns. Although he was aware that these rates could be reduced with the addition of solutes, he decided to explore a different path: that of trying to achieve them by improving heat transfer. Therefore, he was only able to save a small number of tiny biological systems through the process.

It was not until the discovery of glycerol^7^ that these speeds were within what was technically feasible. For this, the dehydration produced by extracellular ice is essential^8,9^. It is the technique known as *slow freezing* that is currently used in thousands of laboratories.

Subsequently, the appearance of other cryoprotectants has led to the development of alternatives to slow freezing. These do not need extracellular ice to achieve the glassification of the cell interior. Due to this complete absence of ice, both inside and outside the cells, they receive the generic name of *vitrification* techniques. In this case, it is common to distinguish between equilibrium and non-equilibrium vitrification, viz. In equilibrium vitrification—after Farrant^10^—increasing concentrations of cryoprotectant are added to the system as its temperature drops, thus mimicking, to a certain extent, the effect of extracellular ice in slow freezing. Given its toxicity^11,12^, the amount of cryoprotectant for each temperature is kept to a minimum, just enough to avoid ice formation, by following the solid–liquid thermodynamic equilibrium diagram; hence its name^10,13–15^. In contrast, in the technique known as non-equilibrium vitrification, all the cryoprotectant is added from the beginning, or in a reduced number of steps, but always, generally, at temperatures above zero and in concentrations capable of reaching the vitrification by simply immersing the sample in liquid nitrogen^16,17^. Obviously, there are intermediate strategies. In them, the concentration of cryoprotectant does not follow the line of the phase diagram, but remains within the bounded band between the possibility of subcooling and the limit of toxicity^3,18^; or one plays with the compatibility of life with a moderate appearance of ice^19,20^. In summary and focusing on what concerns us here: (1) cryopreservation techniques differ from each other fundamentally in the amount of cryoprotectant that the system has for each temperature; (2) said amount of cryoprotectant governs the position, width, and speeds at which this zone in Fig. 1 must be crossed in both directions to avoid ice formation^21^; and (3) in any case, this amount of cryoprotectant is kept to a minimum, given its toxicity. With all this, today, the problem focuses on the optimization of three factors: mass transfer, heat transfer and the toxicity of cryoprotectants. And here is where the geometry and dimensions of the system fundamentally come into play, and consequently, the origin of the difficulty in cryopreserving organs: with small systems, very high cooling/warming rates can be achieved and thus it is possible to keep the concentration of cryoprotectant low^22^. However, when the systems are large, it is more difficult to reach this balance between toxicity and heat transfer, so ice frequently appears in a lethal way, both in those glassified by slow freezing^23^ as by vitrification^4,24^, although generally with very different parameter values, but the origin of the problem is fundamentally the same in the two cases.

Paradoxically, it is easier to avoid the formation of lethal ice crystals during cooling than during warming^21^. The origin of this curious behavior resides in the existence of a double necessity for the appearance of said crystals: that of their nucleation and that of their growth. Because growth requires low viscosity, it occurs preferentially at high temperatures. However, nucleation is most likely at very low temperatures, generally close to Tg (typically $\sim -100 \; ^\circ{\rm C}$ for solutions and $\sim -47 \; ^\circ{\rm C}$ for cells^25^). Thus, when a system cools down, it first crosses the “growth zone”, but for now there is “nothing to grow” (no nuclei yet). Only after, the nucleation zone is reached and small ice embryos may appear that will not grow, and therefore are not lethal in principle. Finally, the system glassifies and it is stored. When it is rewarmed, however, recrystallization (or even devitrification) can occur^26^. Rewarming begins by crossing, and for the second time, the zone of high nucleation, with the possible appearance of new embryos; indeed, Boutron^27^ pointed out the presence of ${10}^{6}$ to ${10}^{12}$ times more nuclei in rewarming from the amorphous state than on cooling from the liquid state. Finally, the growth zone is crossed again, where now there will be a considerable number of opportunistic nuclei that can grow to a lethal size. These crystals, which are more numerous, as we say, during rewarming, see their growth favored as the temperature increases^28^. The way to avoid this growth is *to cross this area very quickly*. That is why a high warming rate is crucial and, in a certain sense, more important than the cooling rate; the relevance of rewarming has recently come to be highlighted as a paradigm shift in cryopreservation^24^. The interested reader can find in the bibliography a deep and detailed analysis of the topics developed in this paragraph^29^.

In systems cryopreserved by slow freezing, as it is this study, as opposed to vitrification, the problem of recrystallization, and the consequent need for high warming rates, has been consistently identified in all sorts of situations. This has led to the recommendation of high warming rates within the Good Manufacturing Processes (GMPs)^30,31^. Thus, it is well known how, in this way, recrystallization is avoided in samples ranging from the size of spermatozoa^32^ to blood bags^30^, even including cryovials in cell therapy^33^. A case of interest has recently been studied in the context of CAR-T^34^, where joint cryomicroscopy and calorimetry studies have identified the benefit of high warming rates to avoid recrystallization.

In order to achieve high speeds of rewarming, different forms of application of the electromagnetic field have been proposed given its penetrating capacity and, therefore, in principle scalable. This application has been done either directly^35–38^ or indirectly through a mediating agent^39–41^. Important advances in this matter have been done recently^42^. A table comparing the various different volumetric heating methods can be found in Supplementary Information.

An alternative to the electromagnetic field, also penetrating^43^, are the High Intensity Focused Ultrasound (HIFU)^44^. The origin of application of HIFU dates to 1927^45^, where they were already talking about it being a field with a wide range of possibilities to investigate. 15 years later the first tumors were being burned by the intense heat generated by these waves^46^. However, it was not until the development of NMR^47^ that imaging techniques allowed HIFU control based on MRI- thermography. Nowadays, there is a wide range of available MRI configurations (sequences) that permit obtaining 3D thermal images for many tissues under different circumstances^48^. On the other hand, it should be noted that the attenuation and penetrability of ultrasound is a function of its frequency (for example, at 1 MHz the ultrasound wave is attenuated approximately 50% as it propagates through 7 cm of soft tissue, and at 2 MHz the wave is reduced to approximately 25% of its initial value by the same tissue^43^). Therefore, for large-sized samples, it is advisable to have an array of transducers, as this enhances the effective wave penetration and scalability by controlling the relative phases of each channel’s wave, dealing with changes in the biomaterial and inhomogeneities (fractures, ice,…) in real time. In this work we used just one transducer (mm-size samples); in previous computer simulations we explored twenty-six transducers (cm-size)^44^. Devices made of thousands of transducers are common nowadays, allowing heating with precision above one tenth of a degree in a multitude of medical therapies^49^.

Our research group recently published^44,50^ the result of computer simulations of HIFU for rewarming in cryopreservation with the promise of putting it into practice in the present work. For this we have used the *C. elegans* as proof-of-principle. Conventional cryopreservation of *C. elegans*^23,51^ is affected by the problem of recrystallization, maintaining low recovery rates in the smallest larval stages—L1 and L2—(35%), and practically zero in the adult stage^51^. However, if rewarming is rapid, this does not occur^52^. In the following lines we show, for the first time, the presentation of HIFU to avoid the problem of recrystallization based on this worm. For this, N2 nematodes were cultivated under usual conditions, at $20\;\mathrm{^\circ{\rm C} }$. With the population stabilized after at least 5 generations, the cryopreservation process was carried out. Two groups were always made for rewarming: a control group, rewarmed in a standard manner^23,51^, and an experimental one, rewarmed with ultrasound. The following paragraphs describe the technical challenges that had to be overcome, the details of the experiments carried out and, finally, the results obtained.

**Figure 2:**
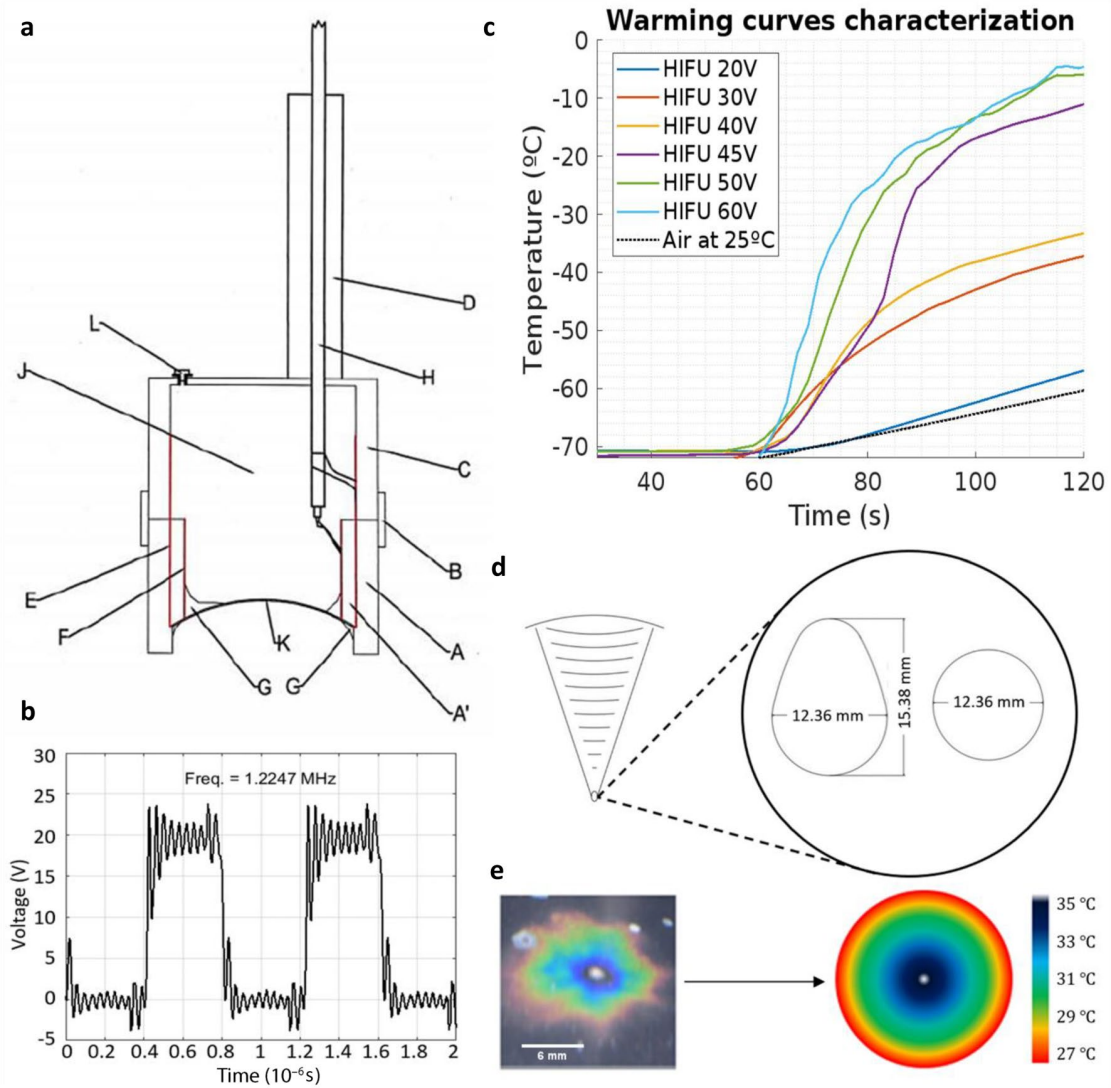
Characterization of the High Intensity Focused Ultrasound equipment. (**a**) Section of the HIFU transducer. A–D: PVC chassis, to provide structure, sealing and air chamber behind the cap; E: conductive film for the outer surface of the cap; F: conductive film for the inner surface of the cap; G: epoxy sealant (external and internal); H: power coaxial cable; J: air chamber; K: PTZ-8 ceramic ball cap; L: drain plug for the air chamber in case of infiltration. (**b**) Square wave generator output, without power supply from the external source. The signal frequency is 1.22 MHz, with an amplitude of 18 V peak-peak. (**c**) Temperature curves obtained by heating with HIFU at different voltages and currents of the external power supply. The shots were normalized for 60 s of exposure. The obtained warming rates depended on the power supply of the external source. As the nominal power of the piezo is 25W, operating below it (case of the curve for 20 V and 0.68 A) results in reduced performance. (**d**) Detail of the focal region of ultrasonic heating. Heat transfer is greatest in an ovoid region of dimensions 12 × 15 mm. These values were obtained from the experiments by thermography with thermosensitive film, whose example is shown in (**e**). (**e**) Thermographic profile of heating with HIFU in water at room temperature. The heating produces, in the section, concentric circles with respect to the focus. Between the hottest and the coldest point, a temperature difference of less than $10 \;^\circ{\rm C}$ is always recorded.

As we mentioned, prior to carrying out the experiments, a series of practical challenges had to be addressed. Thus, a HIFU device was built from a square wave generator using four power transistors with their respective drivers, a 1.22 MHz oscillator, a microcontroller and various passive elements. This wave generator accepts a transistor bridge power of up to 200 W provided by an external source and supplies the excitation to the HIFU transducer. The HIFU transducer is a PTZ-8 spherical cap of 50.8 mm radius of curvature and 10 mm cap depth that is based on the piezoelectric effect to convert square wave electrical excitation into a mechanical oscillation, that is, into pressure waves, with amplitude proportional to the square root of the power used. The cap is in a sealed chamber, described in Fig. 2a. The resonance frequency of the ceramic cap was 1.5 MHz, although it was fed at a lower frequency, as seen in the generator output shown in Fig. 2b, to overcome the possible crossover in the generator transistors. The focus where the ultrasound is concentrated, which depends on the shape and dimensions of the transducer, was determined independently with three different methods: by thermography with a bolometer, by thermography with thermosensitive film, and with thermocouples. The measurements sought to determine the distance on the z-axis that the center of the focal region is located. To obtain precise values, the stepper motors of a FDM CREALITY Ender 3-Pro printer were used to control the position of the thermocouple tip, with an accuracy of 100 µm. The thermal experiments carried out at different distances from the surface of the cap (see Fig. S5) yielded a position of the focus at 50.8 mm with respect to it, this being the point of maximum heating. This is in perfect agreement with what is expected according to the geometry of the transducer detailed above.

With different voltages (in the range of 20–60 V) and currents (in the range of 680 mA to 2.26 A) of DC supply for the wave generator, the heating curves reflected in Fig. 2c were obtained by the transducer, by varying the supply power. The heating rates reached by the device, at the different supply powers, were approximately between 14 and 218 $^\circ{\rm C} /\mathrm{min}$. Next, the shape of the focal region was characterized, as the volume in which at any instant during rewarming, the maximum temperature difference between any two points was always less than $10 \; ^\circ{\rm C}$. This resulted in an ovoid of dimensions 12.4 × 15.4 mm. These values can be seen in Fig. 2d and e.

**Figure 3:**
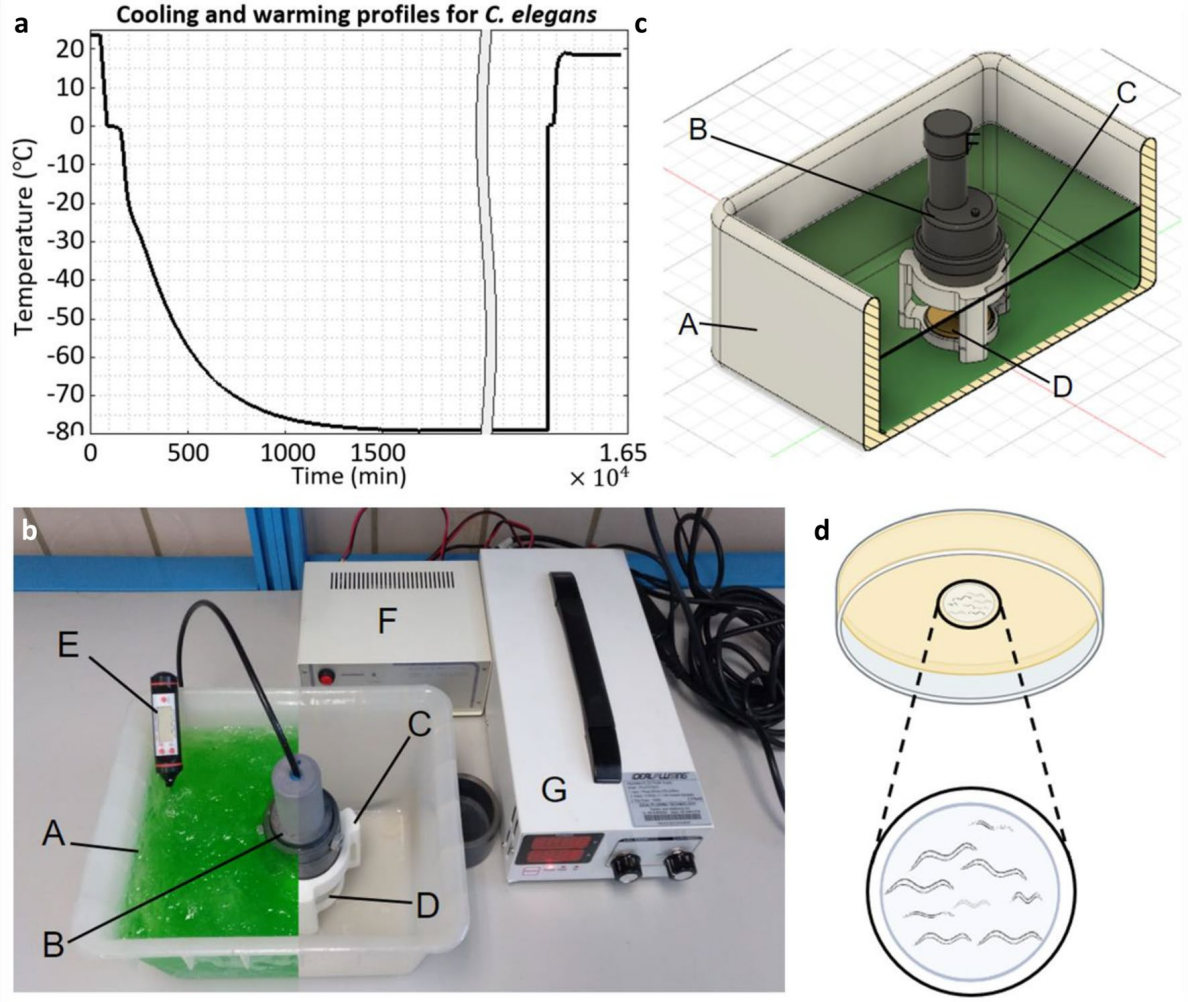
Thermal history for *C. elegans* cryopreservation and experimental setup. (**a**) Temperature curves during the slow freezing process, the storage at $- 80 \; ^\circ{\rm C}$ (represented by the “gap” in the graph) and the recovery by HIFU heating. Samples start at room temperature and, after incubation in cryoprotective solution for 10 min, they are placed in the freezer at $- 80 \; ^\circ{\rm C}$ for a cooling at $- 0.6 \; ^\circ{\rm C} /\mathrm{min}$. Then they are stored for 48 h and recovered by ultrasound heating directly from the storage temperature, up to a temperature of about $-5 \; ^\circ{\rm C}$. (**b**) Experimental setup showing A: ethylene glycol bath at $-70 \; ^\circ{\rm C}$; B: HIFU transducer; C: plate support in PLA; D: Petri dish (with well in its center) covered, sealed with parafilm, and inverted; E: external thermometer; F: square wave generator; G: external power supply. (**c**) 3D section view of the experimental setup, modeled in CAD. In this image, the function of the support and its use is better appreciated. The tags are shared with the image (**b**). (**d**) Detail of the agar well in a 50 mm diameter Petri dish. The plate is presented inverted, which is how it is placed on the 3D support to be exposed to the ultrasound.

In order to guarantee that the entire population of nematodes, soaked in the cryoprotective solution, was confined within the ultrasound focus, they had to be cryopreserved in a small specially-designed container. Said container consisted of a hollow well, built inside a Petri dish, using agar as a filler for the rest. Its detail is shown in Fig. 3d. The dimensions of the well were decided to be 9 mm in diameter by 6 mm in depth, less than the dimensions of the focal ovoid. To ensure the absence of leakage of the cryoprotective medium through the agar, the walls of the well were covered with a film of isopropyl alcohol. The efficacy of the treatment was confirmed by permeability experiments of these wells, revealed by the passage -or not- of phenol red, as staining, through the agar. These custom wells serve merely as a confinement container adapted to our geometry; alternative materials and shapes can also be utilized, as long as they are able to transmit ultrasound. Finally, before cooling, the plates were always closed with their lid and sealed with parafilm. This allows the use of our technology in accordance with the GMPs, as the sample is isolated. For rewarming with ultrasound, ethylene glycol was chosen as the liquid medium for the transmission of sound waves from the transducer to the sample. This medium can maintain nematodes initially at − $80 \; ^\circ{\rm C}$ by remaining in a liquid state even at sufficiently low temperatures.

The necessary immersion of the HIFU transducer by its concave part in the bath frequently generated an undesirable air bubble. These were eliminated by means of a gas sucker submerged in the ethylene glycol. Finally, the repeatability of the experiments and the stability of the position of the focus with respect to the heated sample were guaranteed with the construction of a 3D-printed polylactic acid (PLA) support that securely houses and immobilizes the Petri dish and the transducer, manufactured with CAD and FDM techniques. Figure 3b and c show the experimental setup for the use of HIFU with the manufactured plates. Details on the failure’s modes and the practicality of the heating can be found in the Supplementary Information.

**Figure 4:**
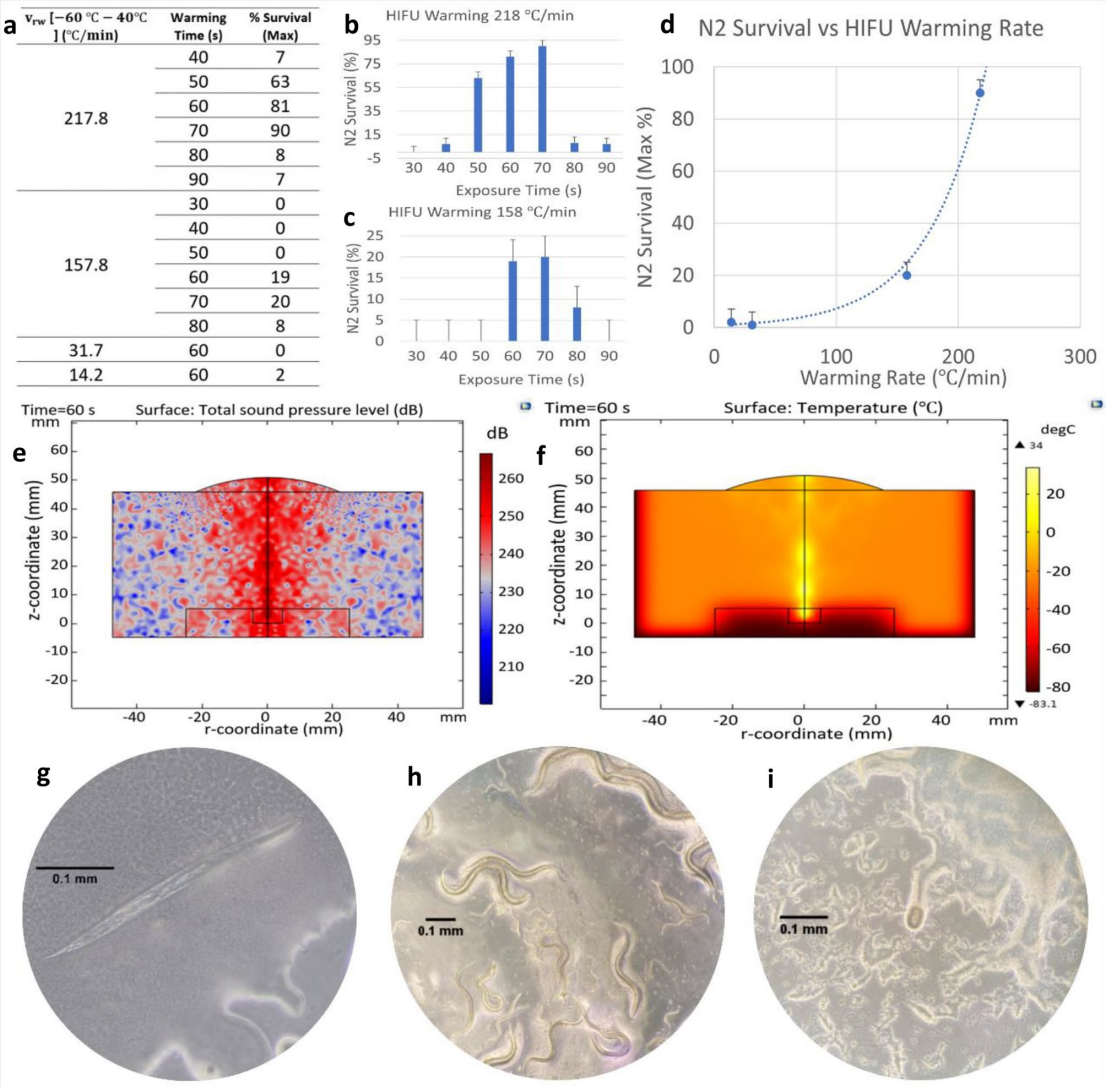
Presentation of results. (**a**) Survival with HIFU according to warming rate and exposure time. Low warming rates show worse results in the recovery of *C. elegans.* The experiments were always done with plates containing groups of $\sim 200$ nematodes. These showed the following average composition: 20% individuals in stage L1, 30% in L2, 25% in L3, 15% in L4 and 10% adults. For the optimal parameters, survival by growth stages was not homogeneous, as more L1–L3 nematodes were recovered than L4 and adults. L4 survival was 70%. Adult survival was between 40 and 60%. (**b**) and (**c**) Show survival for the highest warming rates, $157.8 \; ^\circ{\rm C} /\mathrm{min}$ and $217.8 \;^\circ{\rm C} /\mathrm{min}$ , as a function of exposure time. Short (less than 50 s) and excessively long (more than 70 s) exposure times resulted in very low survival rates. (**d**) Dependence of with the warming rate for the optimum exposure time. For low warming rates the recovery rate is close to 0%, reaching 90% for the highest warming rates. These results must be compared with the Brenner’s protocol, with only 35% for the most favorable stages (L1–L2) and close to 0% for adults. The dashed line is not an interpolation of results, but a trend line. (**e**) Finite element simulation of the acoustic pressure in a 2D section of the experiment. The region of highest acoustic pressure, 270 dB, is reached at the geometric focus of the transducer. (**f**) Simulation by finite elements of the temperature field. in a 2D section of the experiment, after 60 s of exposure to HIFU. The highest temperature is reached at the focus. The Petri dish is placed inverted, with the black rectangle representing the well containing the worms, to avoid any air gap between the ultrasounds and the nematodes. (**g**) Dead nematode after insufficiently rapid rewarming ($<150 \; ^\circ{\rm C} /\mathrm{min}$). (**h**) Nematodes a few hours after being recovered by HIFU heating. All stages of growth are appreciated. (**i**) *C. elegans* egg after the recovery of the nematodes: their reproductive capacity is preserved after cryopreservation and HIFU rewarming.

The physical performance of the entire setup described above was computationally modeled using finite elements (Comsol Multiphysics v6.7), using together the Acoustics and Heat Transfer packages. The result of these computer simulations corroborated both the position of the focus and its dimensions, as well as the warming rates found experimentally. The sound pressure and temperature fields in a 2D view of the experiment are displayed in Fig. 4e and f, respectively. Figure S10 provides an overview of the isothermal contours during a 60 s warming simulation.

After overcoming all the previous details and verifications, we went to the experimental phase with *C. elegans*. For this, the nematodes were cryopreserved following the standard Brenner’s protocol^53^ using slow freezing in 15% glycerol, adapted to our needs. Populations of approximately 200 individuals of all growth stages were cooled at −$0.6 \; ^\circ{\rm C} /min$, up to − $80 \; ^\circ{\rm C}$, within the wells constructed in the Petri dishes described above. The amount of ice formed in this system is discussed in Supplementary Information. After 48 h of storage at − $80 \; ^\circ{\rm C}$ the nematodes were rewarmed. For each experiment there were always two plates. The first plate was rewarmed with ultrasound, applying these until the temperature of the well with the nematodes reached around − $5 \; ^\circ{\rm C}$ (temperature that is above the melting point of the solution). The evolution of the temperature throughout the process is shown in Fig. 3a. The second plate was rewarmed in the standard way: either by leaving it in the air (warming rate: $\sim 10 \; ^\circ{\rm C}$/min) until the solution with the nematodes had completely thawed, or by immersing it in a water bath at $37 \; ^\circ{\rm C}$ (warming rate: $\sim 93 \; ^\circ{\rm C}$/min) for 1 min. After rewarming, the worms were placed on a plate with food. Finally, the recovery of these was observed by analyzing their mobility immediately after depositing them, their mobility after 24 h, and their reproductive capacity.

A total of 53 HIFU experiments involving more than 10,000 nematodes were performed. The table displayed in Fig. 4a shows the survival rate according to the warming rate and the ultrasound exposure time. All warming rates in Fig. 4 were measured in the interval from − $60$ to − $40 \; ^\circ{\rm C}$. This interval was chosen because it is the zone in which the recrystallization problem can be more critical^43,54^.

The ultrasound transducer was powered by four different powers: 13.6 W, 33.9 W, 90.5 W and 134.4 W. Part of this energy was dissipated in the electronics. The associated warming rates, measured by thermocouples, were $14.2 \; ^\circ{\rm C} /\mathrm{min}$, $31.7 \; ^\circ{\rm C} /\mathrm{min}$, $157.8 \; ^\circ{\rm C} /\mathrm{min}$ and $217.8 \; ^\circ{\rm C} /\mathrm{min}$, respectively. Higher velocities could not be explored as the latter is the maximum achievable by our current system.

Prior to the results presented below, a series of preliminary and exploratory experiments were carried out, determining the evidence of the benefit of high warming rates. For this reason, the higher warming rates were later studied in more detail: $157.8 \; ^\circ{\rm C} /\mathrm{min}$ and $217.8 \; ^\circ{\rm C} /\mathrm{min}$. For them a spectrum of exposure times was explored: 30 s, 40 s, 50 s, 60 s, 70 s and 80 s for $157.8 \; ^\circ{\rm C} /min$ and 40 s, 50 s, 60 s, 70 s, 80 s and 90 s for $217.8 \; ^\circ{\rm C} /\mathrm{min}$. The results of these experiments are shown in Fig. 4b and c. In both cases it is observed that insufficient or excessive exposure time to HIFU was fatal for the population under study.

In Fig. 4b, corresponding to a warming rate of $157.8 \; ^\circ{\rm C} /\mathrm{min}$, we can see that for exposures of less than 30 s, 40 s, and 50 s, the recovery rate is 0%. It rises for 60 s, and attains a maximum in 70 s, descending again for longer exposure times, finally reaching 0% (not represented).

Similarly, in Fig. 4c, corresponding to a warming rate of $217.8 \; ^\circ{\rm C} /\mathrm{min}$, for exposures of less than 40 s the recovery rate is 0%. As the exposure time increases to 40 s, 50 s and 60 s the recovery rate gradually increases, reaching a maximum for 70 s. From that value for the exposure time, the recovery rate falls again until it becomes zero.

Finally, in Fig. 4d the survival is plotted against the warming rate for optimal exposure times found previously. This graph shows how the survival rate starts from practically 0% for the lowest warming rates, rises to 20% for intermediate warming rates ($157.8 \; ^\circ{\rm C} /\mathrm{min}$) and finally reaches 90% for the highest warming rates ($217.8 \; ^\circ{\rm C} /\mathrm{min}$).

Regarding adults, for the optimal configuration, $217.8 \; ^\circ{\rm C} /\mathrm{min}$ and exposure time of 70 s, there were 3 plates. In each of them, as in the rest of the plates in this study, there were about 200 worms, of which about 20 were adults. After the application of ultrasound, 7 adults were found in the worst case and 10 in the best of them, representing a survival rate between 40 and 50% for adults, respectively. Videos of the adult recovered worms can be seen in the Supplementary Information (Fig. S11). Remember that the adult recovery rate is close to 0% for conventional slow rewarming.

Figure 4g–i show, respectively, a detail of a nematode killed by an insufficient warming rate, a group of nematodes of all ages after rewarming at $217.8 \; ^\circ{\rm C} /\mathrm{min}$ during 70 s, and an egg laid briefly after nematode recovery.

For completeness, the two forms of standard rewarming, in a bath at $37 \; ^\circ{\rm C}$ and in the air (room temperature: $25 \; ^\circ{\rm C}$), were also registered. In the water bath survival was 0%, which agrees with Brenner’s findings^23^ (see Supplementary Information for discussion of this result). For air, recovery was around 35% for L1–L2 and close to 0% for adults, also in agreement with our recent studies^51^.

We can conclude that these observations prove, for the first time, the ability of HIFUs to achieve warming rates necessary to avoid the problem of recrystallization in cryopreservation. The advantages of this technology are several: (1) it does not generate stationary patterns like microwaves, (2) it does not have the thermal runaway problem, (3) it can be used for samples already stored with conventional cryoprotectants, (4) it can be monitored in real time by MRI, (5) can be easily controlled by interferometry, (6) is routinely used in many other fields, which represents a great benefit, and above all (7) *is scalable*. All this makes this approach a promising tool, either alone or in conjunction with other emerging technologies such as nanowarming^39^. Next possible steps are scaling in the number of transducers, use in conjunction to X-rays computer tomography to monitor the CPA field/ice/fractures, and apply to systems which have already shown some degree of success.

*C. elegans* has been used as an animal model. We think that it is an ideal system, having a complete set of organs, being the basis of important developments in genetics, developmental biology, neuroscience or oncology, and a working tool for more than 500 laboratories around the world. The use of the worm here is for proof of principle only; however, even so, it may be of interest per se for the conservation of the adult state, for strains that freeze poorly or for structures with similar sizes such as the Drosophila embryo^55^. In any case, our technique aids in all situations where the ubiquitous problem of recrystallization may appear, including organ cryopreservation. From its application in this field, we expect a paradigm shift and new ways towards organ and tissue banking.

## Supplementary Information

Supplementary Information 1.

Supplementary Information 2.

Supplementary Legends.

Supplementary Figure 10.

Supplementary Figure 11.

## Data Availability

The datasets used and/or analysed during the current study are available from the corresponding author on reasonable request.
