# Supplementary material for: Sound waves for solving the problem of recrystallization in cryopreservation
Source: Sci Rep. 2023 May 10;13:7603. doi: 10.1038/s41598-023-34681-z (PMC10172391; doi:10.1038/s41598-023-34681-z)
Supplement: Supplementary file 1 — Supplementary Information 1. [file 41598_2023_34681_MOESM1_ESM.docx]

**Supplementary Information**

1. **Maintenance of *Caenorhabditis elegans***

*Caenorhabditis elegans* was maintained as described in the reference protocol [1] and cultured at 20 °C on NGM Petri dishes. Wild-type strain N2 (Bristol) was used. All reagents used were provided by Sigma–Aldrich, except those explicitly indicated below.

- 1. **Preparation of growth media**

*Preparation of bacterial food source:* *C. elegans* was fed with the OP50 strain of *Escherichia coli*. The bacteria were cultured in LB Agar (10 g tryptone, 5 g yeast extract (Labkem), 5 g NaCl, 15 g Agar, H_2_O to 1 L, pH 7.5) in a 90 mm Petri dish (LABBOX, PDIP–E9N–500). A single colony was inoculated in the liquid rich medium L Broth (10 g tryptone, 5 g yeast extract, 5 g NaCl, H_2_O to 1 L, pH to 7.0 using 1 M NaOH) and grown overnight at 37 °C.

*Preparation of NGM Petri dishes:* *C. elegans* was maintained on NGM (Nematode Growth Medium) Agar (3 g NaCl, 17 g Agar, 2.5 peptone (Labkem), 1 ml 1M CaCl_2_, 1 ml 5 mg/ml cholesterol in ethanol, 1 ml 1 M MgSO_4_, 25 ml 1 M KPO_4_, H_2_O to 1 L). The solid components were mixed first in a 2 L Erlenmeyer flask with 975 ml H_2_O and autoclaved for 50 minutes. Once cooled to 55 °C, the liquid components were added (CaCl_2_, cholesterol in ethanol, MgSO_4_, KPO_4_). NGM solution was dispensed in 90 mm sterilized Petri dishes and left at room temperature for 2 – 3 days.

*Seeding NGM dishes:* 0.05 ml of OP50 *E. coli* liquid culture was applied with a 1,000 μl automatic pipette in a 90 mm NGM dish and allowed to grow overnight at 37 °C.

- 1. **Transferring *C. elegans* on NGM Petri dishes**

Once the *E. coli* lawn was prepared, worms were transferred to a fresh dish by chunking This process was repeated until a healthy population of at least five generations was reached before starting the experiments.

The procedures described above are summed up in Figure S1.

1. ***C. elegans* cryopreservation**
   1. **Agar *wells***

To keep the nematodes in a confined volume during the cooling and rewarming processes, Agar *wells* were prepared. Agar was mixed in a glass beaker with distilled H_2_O for a concentration of 24 g/L. The solution was heated up to 80 °C and stirred with a glass rod. The solution was placed in 50 mm Petri dishes (LABBOX, PDIP–E6N–500) and cooled overnight at room temperature (23 °C). Cylindrical holes were then cut into the solid agar with a 1,000 μl pipette tip, thus making the wells. Finally, they were covered with a thin layer of a commercial hair spray based on isopropyl alcohol (Nelly, Spain). The wells had a volume of 200 μl (Fig. S2).

To ensure the watertightness of the well, a test with phenol red was carried out. Phenol red is not just a pH indicator but an intense staining agent, allowing to identify any possible diffusion of the cryoprotectant solution into the Agar.

Petri dishes with and without the isopropyl alcohol lacquer were compared, with the use of lacquer temporally increasing the impermeability of the well and preventing the worms from escaping it. The tests can be observed in Figure S3.

- 1. **Cryoprotectant solution**

S Buffer (129 ml 0.05 M K_2_HPO_4_, 871 ml 0.05 M KH_2_PO_4_, 5.85 g NaCl) was used for the recovery of worms and as carrier of the cryoprotective solution. All reagents were mixed in a 2 L Erlenmeyer flask. K_2_HPO_4_ and KH_2_PO_4_ solutions must be prepared beforehand.

In a 2 ml vial (Simport T311–2), 1 ml of 30% (v/v) glycerol was prepared in S Buffer. It was shaken by inversion twice and put in an incubator at 20 °C for 20 minutes. During the incubation period, 1 ml of S Buffer containing nematodes was prepared in a 2 ml eppendorf. After 20 min, the contents (nematodes in S Buffer) were poured into the vial containing the cryoprotective agent and mixed by inversion twice. The result is 2 ml of 15% (v/v) glycerol solution containing nematodes ready to be cryopreserved.

The procedure described above is summed up in Figure S4.

- 1. **Cryopreservation process**

With a 1000 μl automatic pipette, the Agar wells were filled with 200 μl of the cryopreservation solution containing nematodes. This yields an approximated population of 200 individuals per well. The Petri dishes were sealed with a sheet of parafilm and placed in a polystyrene box modified for our experiments. The maximum cooling rate inside the box was –0.6 °C/min, which ensures slow freezing. The temperature decrease was measured with Type K thermocouples through a PicoLog data logger (Pico Technologies, TC–08). The samples were kept overnight in the – 80 °C ultra-deep freezer (NuAire, UN–6511E).

1. ***C. elegans* rewarming**
   1. **HIFU equipment**

The HIFU equipment is composed of 3 elements: i) a piezoelectric transducer, ii) a wave generator, and iii) a power source. Failure modes analysis can be found in section 4.

**HIFU transducer.** The transducer consists of a spherical cap of 50.8 mm radius, an aperture of 44 mm, and a thickness of 1.56 mm, of a piezoelectric material. The material is lead-titanate-zirconate ceramic (PTZ-8), with a resonance frequency of 1.5 MHz and a piezoelectric behaviour when electrically excited with an oscillating source. A square wave is used for this purpose.

The ceramic is bathed in silver both surfaces to create opposed plates, where the output of the wave generator is electrically bonded. This electrical stimulation creates an electric field between both plates, aligning the ceramic atoms, generating compression and traction. This in turn produces a mechanical wave propagating orthogonally to the surface of the cap.

The transducer has a low impedance (less than 30 Ω). It is encapsulated in a waterproof PVC structure, where the joints are sealed with epoxy resin, to protect the sensitive ceramic and silver coating and guarantee watertightness. An air chamber is formed behind the spherical cap for an efficient and unidirectional propagation of the mechanical wave.

**Square wave generator.** In short, the wave generator (Microaphotec, Spain) consists of high power MOSFETs with their corresponding drivers, an oscillator, and passive elements. The generator accounts for transistor crossover, introducing a slight delay and lowering the switching frequency.

The proposed circuit uses driver ICs (IXYS, IXDD614) for high-power CMOS and fast switching frequency, which feed high currents to the MOS transistors (Vishay-Siliconix, IRF9530). A voltage regulator (Futurlec, 7805T) and various passive elements such as resistors, potentiometers, capacitors, and diodes are used for signal conditioning, decoupling and regulation. Optocouplers are used to isolate the high-power sections of the circuit.

The oscillation is produced by a standard clock (Analog Devices, LTC1799CS5). The circuit also uses a controller (Microchip Technology, ATTINY85S) to start and end the operating cycle, indicated with LEDs.

The generator uses 18V from the source when it is not fed by the power supply. The output of the wave generator was measured with an oscilloscope (Tektronix, TDS 220). A 36V peak-peak square wave was obtained with a frequency of 1.224 MHz, 320 kHz below the resonating frequency of the PTZ-8. This drop in frequency is due to the deliberate introduction of passive elements to prevent crossover in the MOS transistors.

**Power supply.** The power source (IdealPlusing Technology, IPS-ATD10010) provides an adjustable output of 0-100VDC and 0-10A. It consists of two rotary dials, with two potentiometers to regulate the voltage and current output, which are displayed on a screen. Two terminals serve as an output port.

- 1. **Experimental determination of the focal region**

Hereinafter, we will speak of *focal point* when referring to the geometric region in space at which the HIFU waves converge. It is the point of maximum acoustic pressure and where heat transmission is more intense, thus being at a higher temperature than the rest of the heated volume. Considering the spherical cap, the geometric focal region will be the geometric center of the sphere. The theoretical determination of this region is trivial and is given by the design specifications: in our case, a ceramic PTZ-8 with a radius of 50.8 mm.

The rewarming by means of HIFU is carried out in a liquid medium for the correct propagation of the pressure waves. In our case, a mix of roughly 70% ethylene glycol and water is used. This means that the transducer must be immersed, on the one hand, keeping the piezo surface in direct contact with the liquid medium and, on the other, that this medium must be able to remain liquid at temperatures below 0 °C.

To determine the focal point, three experimental methods were employed: i) thermocouples, ii) thermosensitive film, and iii) surface thermography. All three methods involve moving the transducer vertically to obtain the height of maximum and fastest heat transfer.

A precision mechanism with tolerances of less than a millimeter was required to move the transducer along the Z axis. For this reason, a 3D printer (CREALITY, Ender 3) was used, it which the hot end was removed, and the transducer was placed instead. Previously, a fit-to-shape jig was designed and printed to act as a removable clamp holding the transducer, thus replacing the printer's hot end. The clamp was designed in Autodesk Fusion 360 and sliced with CURA. Printing parameters were 0.25 mm layer height, 10% infill, 3 wall thickness profiles, 200 °C hot end in PLA with a 0.200 mm nozzle.

**Focal point determination with thermocouples**: the thermocouple junction was placed at the inferred focal point and ‘shot’ from the vertically standing HIFU transducer for 60 seconds at various heights. The goal is to move the transducer in the z-axis until the fastest temperature rise spot is found. The initial distance was $z_{0}=37.8 mm$ from the base of transducer and increments of 1 mm were added up to $z=47.8 mm$ for a total of 10 steps. See Figure S5 for a reference.

These experiments were performed in water at room temperature (23 °C) using the wave generator without external power supply. Temperature was registered with a data logger (Pico Technologies, TC-08) sampled at 1 Hz.

It was observed that 4 mm away from $z_{0}$, (see Fig. S6), the increase in temperature is the greatest and fastest. This suggests that the focus is located at 41.8 mm ($z_{f}=z_{0}\left( 37.8 mm \right)+ \Delta z\left( 4 mm \right))$ on the z-axis, from the base of the transducer.

Once the gap between the furthest point of the spherical cap and the base of the transducer is computed ($\sim$10 mm), a total distance of 50.8 mm is obtained, which is equal to the transducer radius R, thus corroborating the geometrical focal point. This is shown on Figure S5.

**Focal point determination with thermosensitive film.** This is achieved by observing the change in colour and size of the imprint that the transducer leaves on thermosensitive film while active. The film was placed at the bottom of a 2 L glass beaker, filled with water and inside which the transducer was moved along the z-axis.

The film has an operating range of 27 to 35 °C, being completely black for temperatures equal to or less than 27 °C, and turning red, yellow, green, and blue as it approaches 35 °C, saturating in intense dark blue at this temperature.

The incidence of the focused ultrasound damages the thermosensitive paper near the focal point, so it follows that the temperature at that point is higher than that which the paper can record.

Qualitatively, the size of the coloured mark is measured for each increment $\Delta z$ on the vertical axis. The greatest dimensions were obtained between $\Delta z=3 mm$ and $\Delta z=4 mm$, for a focal region centre at approximately $z_{f}=41 mm$ from the base of the transducer. This is consistent with the data obtained with the thermocouples.

**Focal point determination with a bolometer.** Surface thermography was performed with a bolometer. The main drawback of this method is the type of measurement, which is superficial, while the HIFU transducer creates a warming volume.

To solve this problem, the thermosensitive element (infrared camera) was placed under the base of the beaker filled with water at 22 °C. Thus, by moving the transducer along the z-axis, the temperature at the floor surface of the beaker can be measured for each height increment $\Delta z$.

The water inside the beaker was replaced after each recording to prevent the water from warming up gradually through the experiments. An average temperature is computed for the whole 40x40 pixel matrix and thermograms are stored after 60 seconds of exposure to HIFU.

The greatest temperature increase was reached at $\Delta z=4 mm$, or $z_{f}=41 \mathrm{mm}$, which is consistent with the results obtained from the previous methods.

The following conclusions were drawn from the three methods:

1. The focal region center is placed at 41.8 mm in the z-axis from the bottom of the transducer, as determined with all methods, but mainly and most reliably with thermocouples.
2. The focal region has an ovoid-like shape of 12.4 mm diameter in the OXY plane and a height of 15.4 mm, as determined by surface thermography.
   1. **Design of a 3D printed plastic stand**

Ensuring all rewarming experiments are performed at a set distance, equal to the focal distance, was imperative. A custom stand was designed with CAD (Autodesk Fusion 360) to allow repeatability. Several iterations were carried out to adjust the stand to the needs of the experiment involving Petri dishes.

The 3D stand has a base on which to place the Petri dish, three pillars to provide stability and support the clamping ring, where the transducer rests with a tight fit. The vertical pillars maintain the desired focal distance, 41.8 mm from the base of the transducer. The dimensions can be observed in Figure S7.

This device was designed so that it could be used inside a 1 L capacity beaker and other containers with a flat floor. The stand was printed in PLA with an FDM printer (CREALITY, Ender 3) after being sliced (Ultimaker, CURA) for a layer height of 200 μm, a nozzle temperature of 200 °C, bed temperature of 60 °C, 3 perimeter walls and supports.

- 1. **Rewarming procedure**

This protocol is transversal to all our nematode experiments involving ultrasounds. Before warming the samples, preparatory ‘shots’ were carried out in the ethylene glycol bath without any biological sample inside. This ensures the operating voltage and current are being used. The power drawn by the system is dependent on the medium in which the transducer is immersed. Experiments were carried out feeding the generator with different values for the power source. Warming rates from 14 °C/min to 218 °C/min were achieved at voltages between 20V and 60V DC. The effect of the warming rate on nematode recovery was studied.

The Petri dishes are placed on the 3D support, facing down, to minimize sound wave reflection by making the air trapped inside the dish face away from the transducer, and thus having the well and nematodes be directly exposed to the ultrasounds. This also ensures that the thawed nematodes are trapped by the dish’s cover and not lost as they diffuse through the ethylene glycol. See Figure S2 for reference. A thin layer of polycarbonate stands between the sample inside the well and the medium through which the ultrasounds propagate. This, although creating partial reflection of the mechanical waves, is sufficiently thin for heat to be properly transferred through it and the samples to warm up, as was shown in finite-element simulations.

A Petri dish with *C. elegans* is recovered from the –80 °C ultra-deep freezer. It is rapidly placed on the plastic stand as stated above. The stand is then submerged in ethylene glycol at –40 °C. Ultrasounds are ‘shot’ at the dish for different exposure times, from 30 to 90 seconds. This yields different final temperatures in the sample.

Finally, the dish is removed from the plastic stand and viability is assessed. When working with the dish upside down, nematodes may be found both in the Agar well and the dish cover as they precipitate during thawing. Nematodes were extracted with S Buffer and a 1,000 μl automatic pipette and placed in an NGM Petri dish with OP50 strain *E. coli* lawn.

Viability tests were assessed both immediately and the following day considering all growth stages, visually inspecting motility. Reproductive capacity (consecutive generations of nematodes) was evaluated after 24 hours by counting eggs laid and new larval-stage individuals.

1. **Failure Modes**

Research groups interested in implementing HIFU in their laboratories should acknowledge the following indications:

1. HIFU requires acoustic impedance matching with the wave propagation medium to avoid wave reflection and inefficiencies, but also to prevent the overheating of the device due to bubbles being formed in the concave surface of the spherical cap.
2. Air-backed transducers are recommended, as it forces one directional wave propagation, enhancing the acoustic pressure put on the sample and improving efficiency.
3. In relation to 2), water tightness is crucial and a potential source of failure.
4. Sound waves can reflect off of surfaces depending on density, angle of attack, etc. This should be closely inspected before further application to samples of interest. This has been only observed with polymers thus far.
5. Penetration depth decreases with frequency. At 1 MHz, the ultrasound wave is attenuated approximately 50% as it propagates through 7 cm of soft tissue. At 2 MHz, the wave is reduced to approximately 25% of its initial value in the same tissue.
6. If open-loop HIFU warming, i.e non-guided HIFU, were to be used, calibration steps are required before the warming is carried out on samples of interest. This calibration protocol is entails experimental (additionally to theoretical) thermal focal point determination, warming rate measuring on phantoms physically similar to the sample of interest, and power selection.

**Ice formation within the sample**

Slow freezing allows the formation of extracellular ice, provided that intracellularly, it is not tolerated. For a solution containing 15% v/v glycerol in KHB, estimations can be made taking Peter Mazur’s data on the unfrozen fraction at various temperatures given the solute concentration of glycerol and NaCl. Our calculations yield an unfrozen fraction of $0.113 (11.3\%)$ at $-80 ℃$ as it is shown in Figure S12.

**Supplementary Tables**

| Technology | Nanowarming | Dielectric | HIFU |
| --- | --- | --- | --- |
| Warming rate ($\boldsymbol{℃/min}$) | $\geq{10}^{2}$ | ${\geq10}^{2}$ | ${\geq10}^{2}$ |
| Warming volume (mL) | Scalable | Scalable | Scalable |
| Homogeneity, $\boldsymbol{\Delta T (}\boldsymbol{℃}$) | Achievable by controlling uniform distribution of nanoparticles | Need of controlling thermal runaways; Frequency dependent | Attenuation dependent |
| Penetration (cm) | EM field dependent | Material (CPA and tissue) dependent | Frequency dependent |
| Type of Solution | CPA and Nanoparticles | CPA only | CPA only |
| Need of physical  coupling media | No | No | Yes |
| Real time control | Not required | Not required | Required (MRI) |

**Table 1:** Comparison between three technologies for warming in cryopreservation. Nanowarming employs ferromagnetic nanoparticles inserted into the sample that are warmed up due to hysteresis in a coil-induced alternating electromagnetic field. Dielectric warming is based on the principle of radiowaves and the dielectric potential of dipolar molecules, i.e. water, in the sample. HIFU employs acoustic waves that propagate towards a single, focused spot that is heated up due to the vibration of molecules. In the first two rows (achievable warming rate and scalability), all three technologies fall within the desirable range, especially when compared to convective heating (standard water bath). However, they differ significantly in the remaining aspects, each with its own advantages and disadvantages. To highlight the major drawbacks of each, we have: for nanowarming, the need to use nanoparticles; for dielectric heating, the difficulty of controlling thermal runaways; and for HIFU, the need for a medium that serves as a physical coupling agent and the need of real-time control through imaging techniques (MRI)..

| $\boldsymbol{Initial Temperature}$ | $\boldsymbol{Cooling rate (℃/min)}$ | | | | |
| --- | --- | --- | --- | --- | --- |
|  | $[-196 ℃-140 ℃]$ | $[-140 ℃-80 ℃]$ | $[-80 ℃-60 ℃]$ | $[-60 ℃-40 ℃]$ | $[-40 ℃ 0 ℃]$ |
| $\boldsymbol{T}_{\boldsymbol{o}}\boldsymbol{=-}\boldsymbol{80}\boldsymbol{℃}$ | $N/A$ | $N/A$ | $400$ | $218$ | $85$ |
| $\boldsymbol{T}_{\boldsymbol{o}}\boldsymbol{=-}\boldsymbol{140}\boldsymbol{℃}$ | $N/A$ | $480$ | $300$ | $190$ | $70$ |
| $\boldsymbol{T}_{\boldsymbol{o}}\boldsymbol{=-}\boldsymbol{196}\boldsymbol{℃}$ | $611$ | $225$ | $150$ | $100$ | $40$ |

**Table 2:** Cooling rate comparison through finite-element analysis. Warming rates have been measured in 5 temperature intervals, for three different storage temperatures. The warming rate decreases as temperature increases due to the acoustic attenuation.

**Supplementary Figures**

**
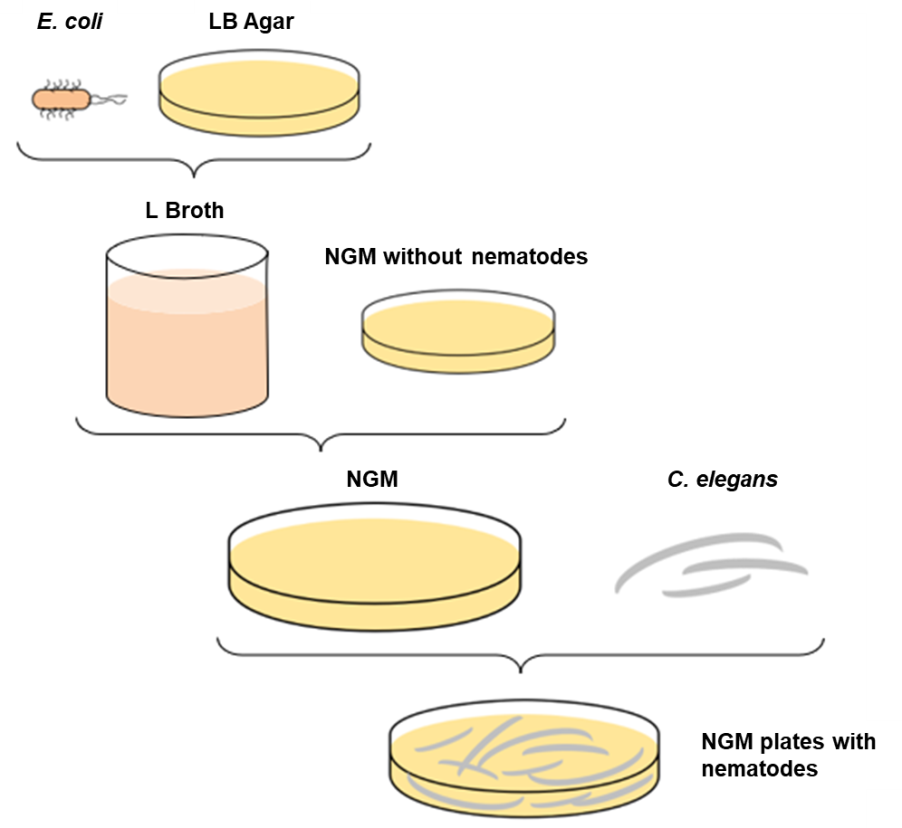
**

**Supplementary Figure 1.** *C. elegans* maintenance diagram. OP50 strain *E. coli* was fed to the nematodes. One colony was inoculated in L Broth rich medium. Once the desired bacterial growth was reached, samples were deposited on NGM, the culture medium for nematodes. By chunking, the worms were transferred to the new NGM dish, and their growth was monitored.


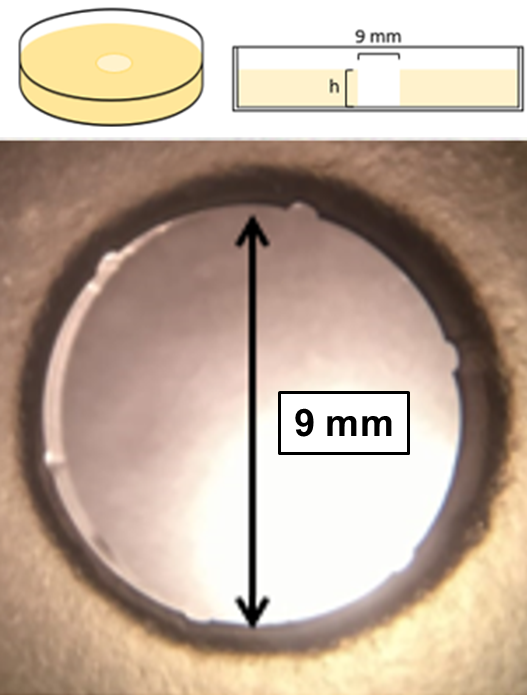

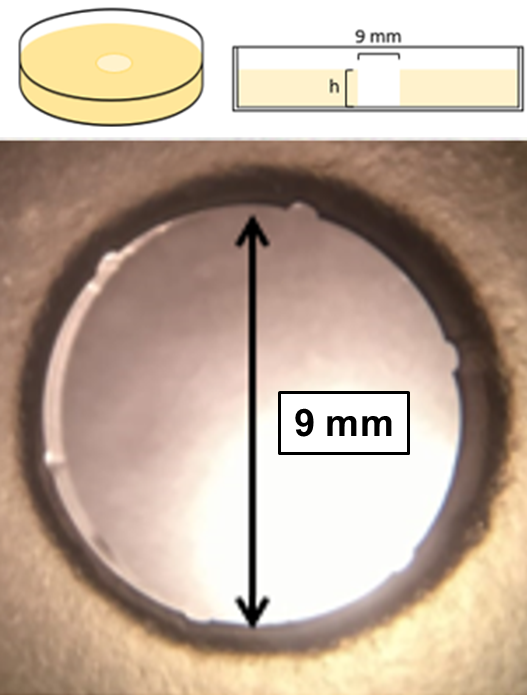

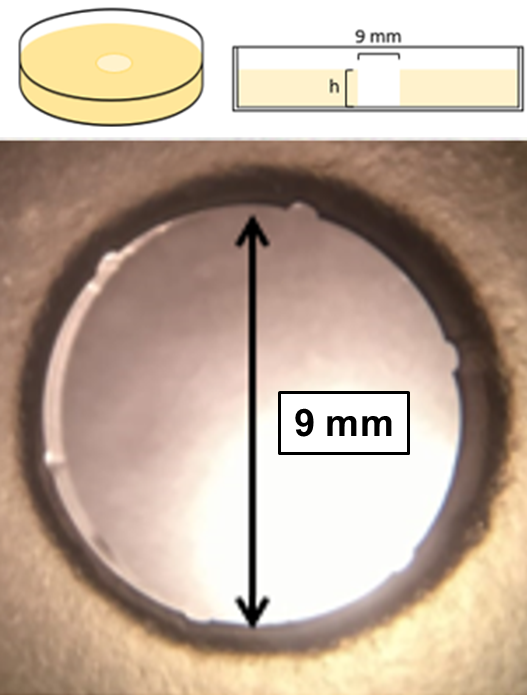


**Supplementary Figure 2.** Agar wells in detail. 24 g/l Agar solution was poured into 50 mm dia. Petri dishes. Once the content solidified, the well was cut into it with a 1,000 μl pipette tip. Finally, a layer of isopropyl alcohol lacquer was placed to make the hole impermeable. The well was 9 mm diameter in diameter with h = 5 mm depth and a volume of 200 μl.


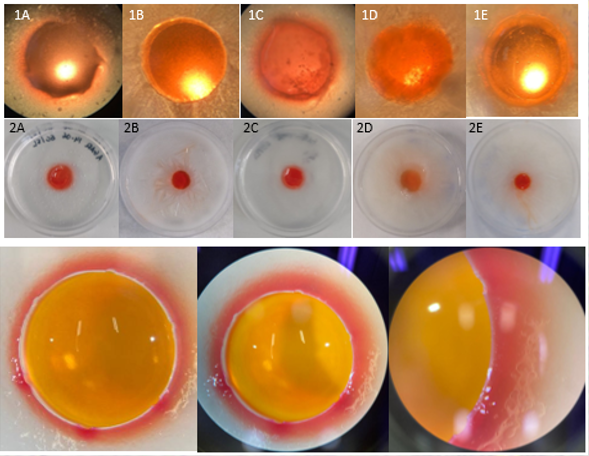


**Supplementary Figure 3.** Diffusion tests with phenol red as a dye and isopropyl alcohol lacquer as a waterproof coating. A solution of phenol red in sodium hydroxide (1M NaOH) was prepared to dye the cryoprotectant solution. Wells were tested for solution diffusion with and without coating both on frozen and unfrozen wells. Images 1A – 1E are details of images 2A – 2E under a loupe (OPTIKA SMZ-2, SN 387707). **1A**, **2A**, no lacquer, unfrozen; **1B**, **2B**, no lacquer, frozen; **1C**, **2C**, lacquer, unfrozen; **1D**, **2D**, lacquer, frozen after coating; **1E**, **2E**, lacquer, frozen before coating. The result of this test yielded: i) that the isopropyl alcohol lacquer offers an impermeable layer, at least temporarily, against the penetration of the cryoprotective medium into the agar. This is verified by the shallower depth of penetration in wells 1C and 2C compared to 1A and 2A; ii) that freezing causes cracks in the Agar due to the stress inherent in the frozen solid sample. This can be seen in dishes 2B, 2D and 2E; iii) that cracks formed during freezing can be partially covered by spraying the lacquer. However, although this option (1E, 2E) is better than freezing the dish after coating (1D, 2D), it is still not as desirable as coating before freezing (1C, 2C). After one hour at room temperature (around 22 °C), the solution began to diffuse through the lacquer coating, into the Agar in all dishes. Coated wells, however, were permeated in delay and at a slower rate. In conclusion, applying isopropyl alcohol lacquer before placing the nematodes prevents the permeation of the cryoprotective solution in the first moments of its cryopreservation, where the solution is less viscous, and confines the nematodes until slow freezing is in an advanced stage.

**
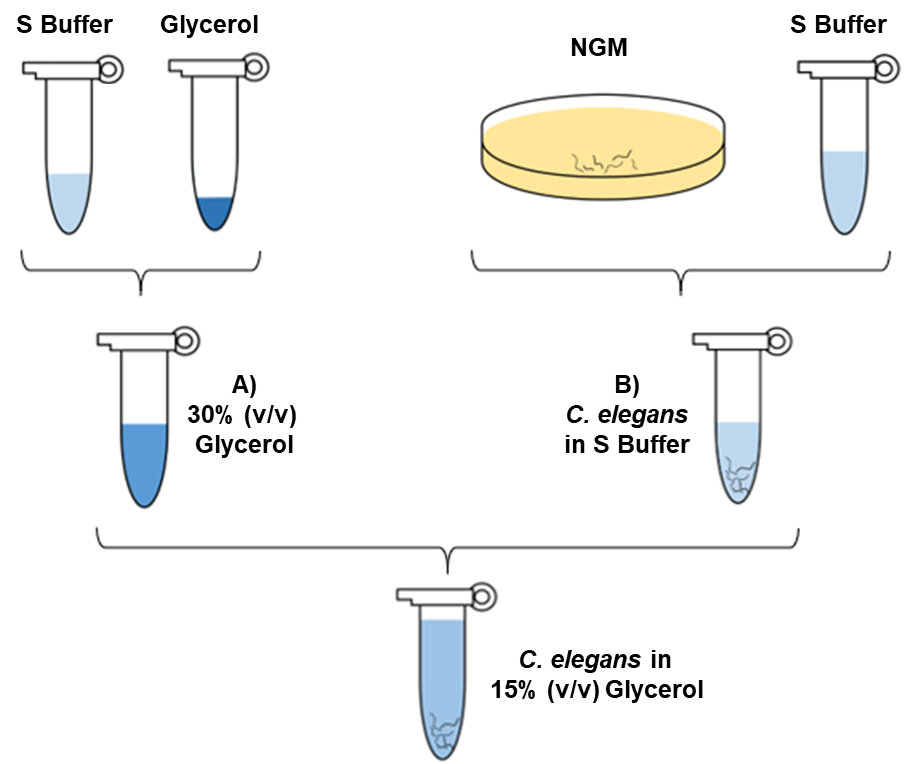
Supplementary Figure 4.** Preparation of the cryoprotective solution. The process involves two solutions: A) 30% (v/v) glycerol in S Buffer (1 ml) and B) 1 ml with worms immersed in S Buffer (1 ml). A) was incubated for 20 min at 20 °C before inversion-mixing with B). The result was the 15% (v/v) glycerol solution, the conventional cryopreservation protocol for *C. elegans* [1].


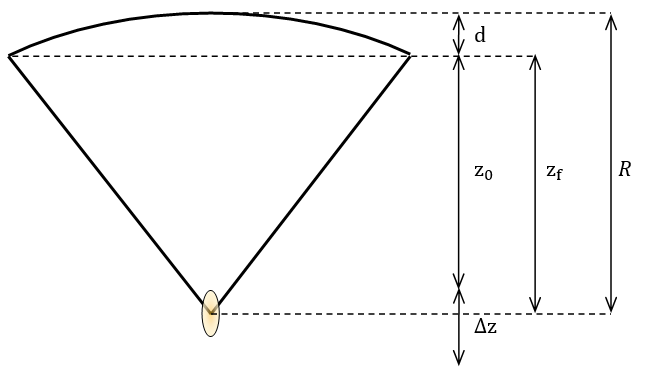


**Supplementary Figure 5.** Determining the distance of the focal region centre. The diagram shows the spherical cap concentrating the waves in one small region. $d$: spherical cap depth, $\sim10 mm$; $z_{0}$: initial distance, $37.8 mm$; $z_{f}$: focal distance, to be determined; $\Delta z$: distance increments, experimental range for focal point location; $R$: real focal point location, at radius $R=50.8 mm$.


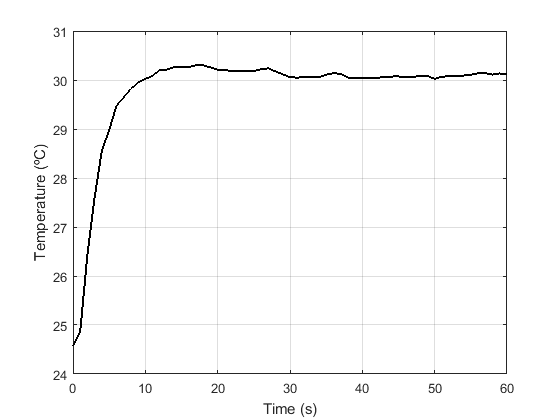


**Supplementary Figure 6.** Experimental characterization of the focal region using thermocouples. This distance of 4 mm yielded the fastest ($t_{rise}=10 s$) and greatest ($\Delta t\approx6 ℃$) temperature increase. Note the temperature increase was observed in water at room temperature ($22 ℃$). Greater increases are obtained at sub-zero temperatures.


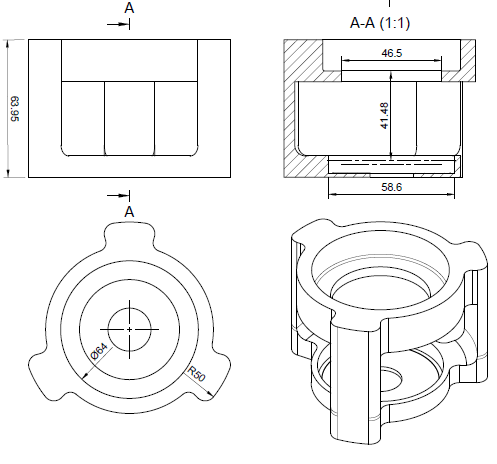


**a b**

**c d**

**Supplementary Figure 7.** 3D printed plastic stand to hold the transducer in place. **a**: front, **b**: top and **c**: section views, as well as **d**: isometric view is presented. White PLA was used for this print. A whole was cut at the bottom to easily remove the Petri dish once the experiment has been carried out.


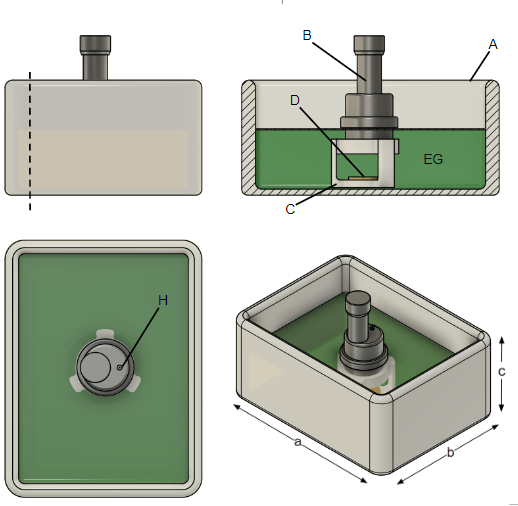


**Supplementary Figure 8.** Four different views of the experimental setup. In the upper left, the dashed line represents the cut shown in the upper right view. The capital letters in the upper right indicates: A: Plastic container with all the elements. B: Ultrasound transducer. C: Stand for holding the Petri dish and the ultrasound transducer. This stand guarantees the precision positioning of the focus on the worms D: Petri dish containing the worms. This Petri dish is placed in an inverted position in the stand. In that way, the ultrasound focus covers the sample without crossing any possible air layer. EG: Ethylene glycol bath for the propagation of the ultrasound. A gradient in temperature exists from the surface (room temperature, 23 °C) till the bottom (– 70 °C). This gradient in temperature generates a gradient in viscosity. In that way, the change in the acoustic impedance is smooth in the propagation media, avoiding reflections. Lower left: represents an upper view of the setup. The letter H indicates a small hole with a rubber plug existing in the transducer handle. Its mission is for draining any liquid that could eventually enter this cavity. The lower right panel shows a 3D view of the complete setup: a= 288 mm; b= 215 mm; c= 142 mm.


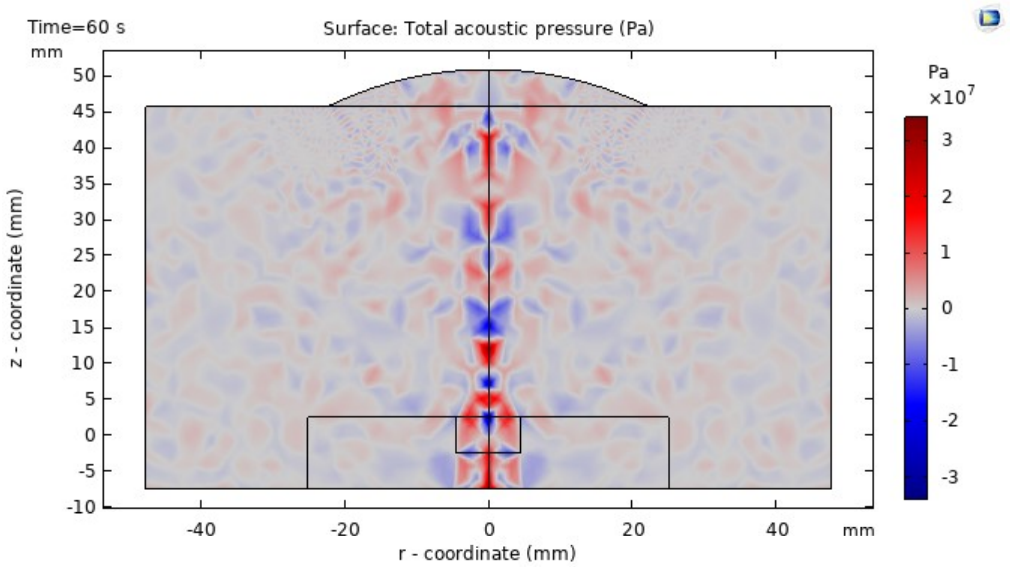


**Supplementary Figure 9.** Finite element simulation showing the acoustic pressure (Pa) profile of a 2D cut plane in HIFU rewarming of cryopreserved nematodes. The transducer is the curved shape at the top. The larger rectangle represents the ethylene glycol bath, and the smaller ones are the Petri dish filled with Agar and the well containing the cryopreserving solution. The transducer produces high- and low-pressure spots of increasing value, the point of highest acoustic pressure being at the focal point. Recrystallization occurs primarily in regions where the viscosity is low enough to allow for ice crystal growth. It is in these areas of relatively low viscosity that it becomes essential to have high rewarming rates, as achieved through HIFU in this case.


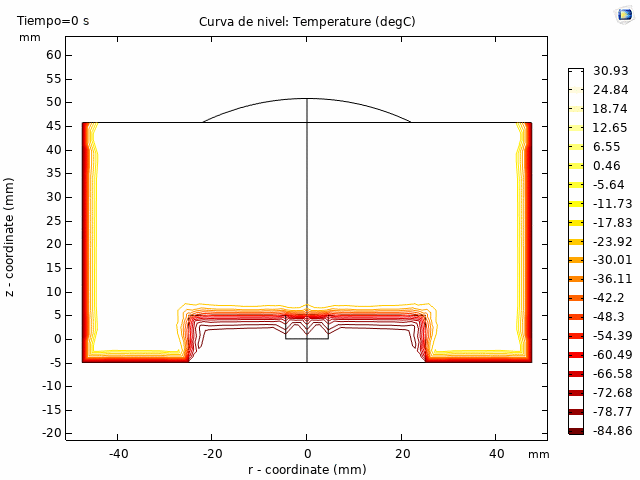


**Supplementary Figure 10**. Finite element simulation showing the isothermal contours of a 2D plane cut of the experiment. The sample is initially at –80 °C, the surrounding ethylene glycol is at –70 °C. The transducer is simulated to be turned on at t = 5 seconds. The acoustic waves warm up the medium, the point of highest energy transfer being at the focal point. The sample is heated up to $\sim$0 °C after roughly 1 minute of exposure to ultrasounds.


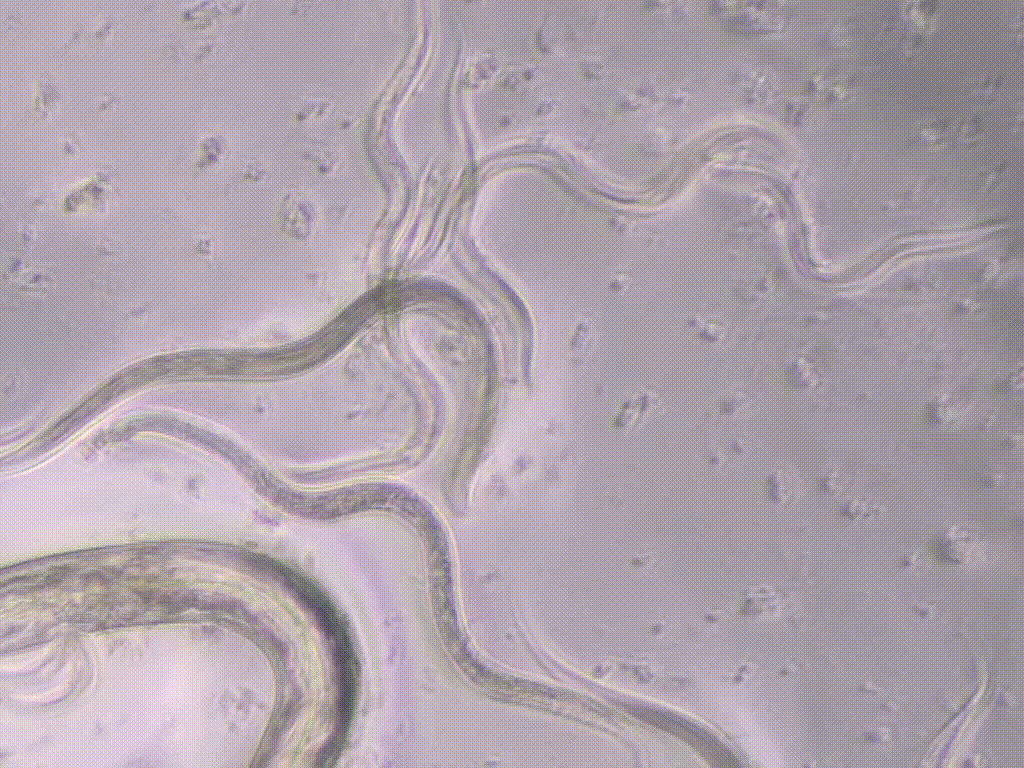

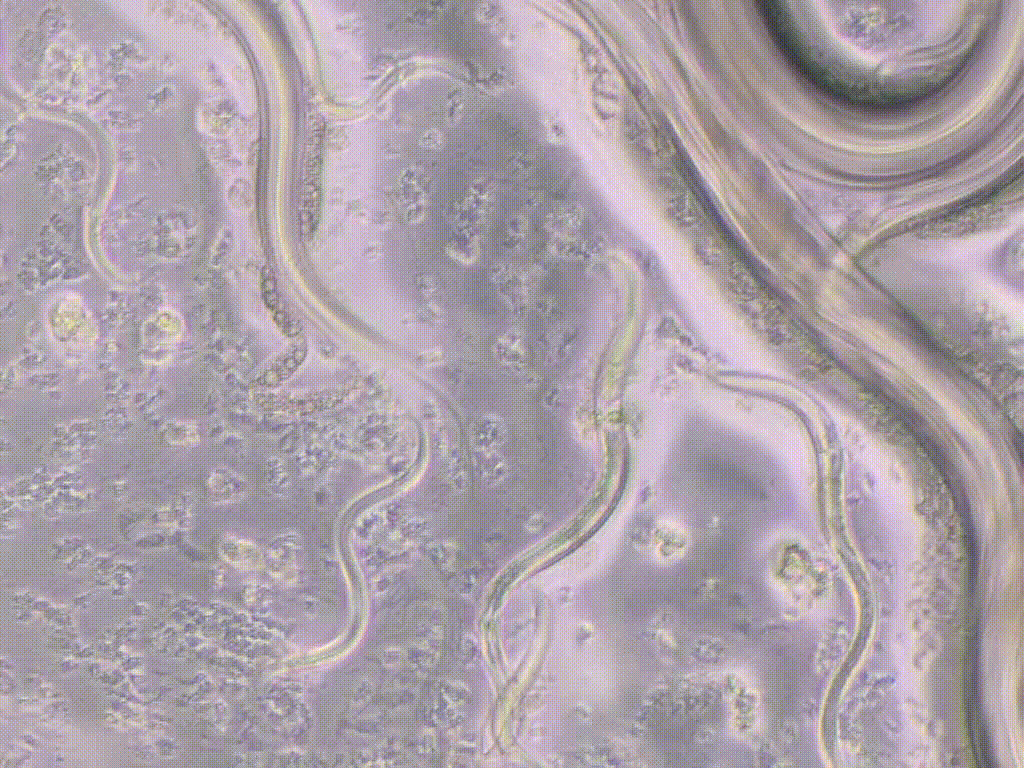


**Supplementary Figure 11**: Nematodes after rewarming with HIFU. Both GIFs represent nematodes of all growth stages a few hours after being recovered from – 80 °C using ultrasounds. Several adult worms are shown.


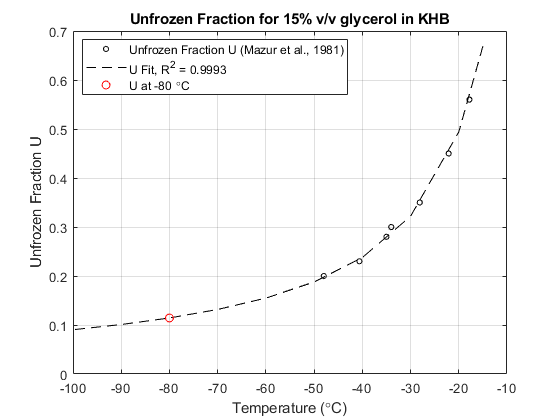


**Supplementary Figure 12**: Unfrozen fraction as it varies with temperature for a 4 M glycerol in KHB. Data was taken from Mazur’s work on the freezing of living samples in 1981 [2]. The original data is obtained with a $0.6 ℃/min$ cooling rate. Curve fitting was done to the data and a point was extrapolated for an approximated unfrozen fraction at $-80 ℃$. This yields an unfrozen fraction of 0.113, or 11.3% of the sample. Note that the corresponding nematode volume should also be subtracted from the final frozen volume.

**Supplementary References**

- 1. Stiernagle, T. Maintenance of C. elegans. *WormBook*, 1-11 (2006).
  2. Mazur, P. Principles of medical cryobiology: The freezing of living cells, tissues, and organs. In: Bittar E., & Bittar N. (eds). Principles of Medical Biology. Elsevier 4D, 355-384 (1996)
  3. Culjat MO, Goldenberg D, Tewari P, Singh RS. A review of tissue substitutes for ultrasound imaging. Ultrasound Med Biol. 2010 Jun;36(6):861-73. doi: 10.1016/j.ultrasmedbio.2010.02.012. PMID: 20510184.
