# Supplementary figures and images for: Sound waves for solving the problem of recrystallization in cryopreservation
Source: Sci Rep. 2023 May 10;13:7603. doi: 10.1038/s41598-023-34681-z (PMC10172391; doi:10.1038/s41598-023-34681-z)

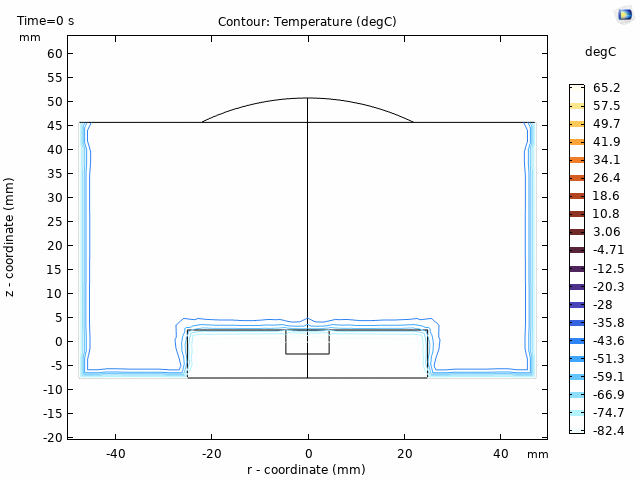

Supplement: Supplementary file 2 — Supplementary Information 2. [file 41598_2023_34681_MOESM2_ESM.gif]
