# Supplementary material for: Sound waves for solving the problem of recrystallization in cryopreservation
Source: Sci Rep. 2023 May 10;13:7603. doi: 10.1038/s41598-023-34681-z (PMC10172391; doi:10.1038/s41598-023-34681-z)
Supplement: Supplementary file 3 — Supplementary Legends. [file 41598_2023_34681_MOESM3_ESM.docx]

a) The file “Isothermal Contours.gif” corresponds to Fig 10 of Supp Info. Its legend is:

**Supplementary Figure 10**. Finite element simulation showing the isothermal contours of a 2D plane cut of the experiment. The sample is initially at –80 °C, the surrounding ethylene glycol is at –70 °C. The transducer is simulated to be turned on at t = 5 seconds. The acoustic waves warm up the medium, the point of highest energy transfer being at the focal point. The sample is heated up to $\sim$0 °C after roughly 1 minute of exposure to ultrasounds.

b) The files “Worms after ultrasound rewarm 1.mov” and “Worms after ultrasound rewarm 2.mov” corresponds to Fig 11 of Supp Info. Its legend is:

**Supplementary Figure 11**: Nematodes after rewarming with HIFU. Both GIFs represent nematodes of all growth stages a few hours after being recovered from – 80 °C using ultrasounds. Several adult worms are shown.
